# Immunomodulatory properties of Giloy (*Tinospora cordifolia*) leaves and its applications in value-added products

**DOI:** 10.1016/j.heliyon.2024.e40948

**Published:** 2024-12-07

**Authors:** Jyoti Singh, Etika Saxena, Anjali Raj Chaudhary, Mandeep Kaur, Molly Salotra, Prasad Rasane, Sawinder Kaur, Sezai Ercisli, Melekber Sulusoglu Durul, Mehmet Ramazan Bozhuyuk, Ahmet Hakan Urusan, Riaz Ullah

**Affiliations:** a Department of Food Technology and Nutrition, Lovely Professional University, Phagwara, Punjab, 144411, India; b Department of Horticulture, Faculty of Agriculture, Ataturk University, 25240, Erzurum, Turkiye; c Department of Horticulture, Faculty of Agriculture, Kocaeli University, 41200, Kocaeli, Turkiye; d Department of Horticulture, Faculty of Agriculture, Igdir University, 76100, Igdir, Turkiye; e Department of Plant and Animal Science, Vocational School of Food, Agriculture and Animal Science, 12200, Bingol, Turkiye; f Department of Pharmacognosy, College of Pharmacy, King Saud University, Riyadh, Saudi Arabia

**Keywords:** Giloy leaves (*Tinospora cordifolia*), Immunomodulatory, Functional food, Phytochemical composition, NF-κB pathway, Interleukin modulation, Antioxidant activity, Apoptosis regulation, Value-added products

## Abstract

In Ayurvedic texts, Giloy (*Tinospora cordifolia)* have been known as the most potent and important medicinal product. Giloy leaves have been used for centuries to cure various ailments and diseases in the human body. This review highlights that giloy leaves have immunomodulatory properties and can be used to develop functional food products. The current review focuses on the phytochemical composition of giloy leaves, and the mechanism for their immunomodulatory action highlighting specific pathways including NF-kB pathway and interleukin modulation. Giloy leaves possess antioxidant activity and induce apoptosis of cells in the immune response. Studies have shown the potential of incorporating giloy leaves into a wide range of value-added products, such as beverages, biscuits, and herbal formulations, to bridge traditional knowledge with modern functional food production. A comparison of traditional and modern giloy-based products demonstrates their benefits and drawbacks, showing that traditional techniques can be combined with modern scientific developments to improve medicinal efficacy. Although giloy leaves-based products show promising potential, further research is necessary to ascertain their effectiveness.

## Graphical abstract

**Figure:**
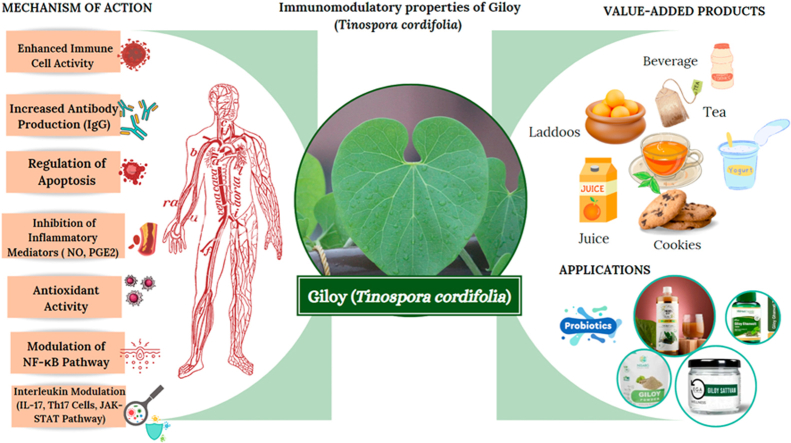

## 1 Introduction

Medicinal plants have been an essential component of conventional health care around the world for ages since Ayurveda [1]. According to these natural resources provide a wide range of chemicals that are bioactive with a variety of therapeutic potential. *Tinospora cordifolia*, often called Amrita or Guduchi, holds a great significance in Ayurveda owing to the presence of various secondary metabolites [2,3]. Giloy leaves have historically been utilized to cure a variety of ailments, such as diabetes, rheumatism, jaundice, and skin diseases [4,5]. Because of its historical use and the increasing popularity of natural therapies, scientists are looking into the possible therapeutic uses of giloy leaves and the underlying mechanisms behind their medicinal action [6].

Extract of the giloy leaves helps to alleviate the digestive ailments such as acidosis, gastroenteritis, parasitic infections, lack of appetite, stomach discomfort, extreme thirst, and nausea, as well as liver-related issues like hepatitis [7].

The latest study indicates that giloy leaves immunomodulatory capabilities may be associated with its numerous potential uses. These characteristics may strengthen the immunological response within the body, providing a natural means of promoting general well-being [8]. Despite having a wide range of uses in traditional medicine, the immunomodulatory effects of giloy leaves is less known [9].

This review aims to reduce this gap by thoroughly analyzing immunomodulatory potential of giloy leaves. The research findings about its capacity to regulate the immune system, with particular attention to important pathways such as NF-κB signalling, inflammatory regulation, induction of apoptosis, phagocytosis stimulation, and interleukin modulation. Giloy leaves have been used in the creation of functional food products by comprehending these processes. Through the integration of contemporary scientific findings with traditional wisdom, our goal is to provide insight into the immunomodulatory mechanisms of Giloy leaves and their potential to enhance overall health and well-being. Then, by utilizing this knowledge, innovative giloy leaves-based functional food products can be made, bridging the gap between conventional wisdom and current medical developments.

## 2 Methodology

### 2.1 Identifying relevant research

A systematic search of significant electronic databases such as PubMed, Scopus, and Google Scholar has been done to thoroughly examine the immunomodulatory activities of Giloy (*Tinospora cordifolia*) leaves. The search strategy included various terms that addressed multiple aspects of immune system regulation and prospective uses. Terms such as “*Tinospora cordifolia*” or “Giloy leaves” were used here, along with terms on general immunomodulatory effects, cellular mechanisms of the immune response, and product development, such as “cytokines,” “macrophages,” and “natural killer cells,” as well as terms related to immune system function and anti-inflammatory properties. This comprehensive strategy intended to collect all relevant data on how *Tinospora cordifolia* affects the immune system and to investigate the potential use of giloy leaves in the development of future functional food products.

### 2.2 Inclusion and exclusion approach

The review process involves a two-tiered selection procedure with a primary focus on research that clarifies the mechanisms of action by which giloy (*Tinospora cordifolia*) leaves affect the immune system. The featured research examines how *Tinospora cordifolia* affects immune cells or function in animal models or in vitro (lab-based), is published in scholarly journals that undergo peer review, and provides clear evidence of *Tinospora cordifolia's* action mechanisms on immunological function, including the involvement of specific bioactive chemicals extracted from its leaves.

The research will focus on the utilization of *Tinospora cordifolia* in value-added products, particularly in functional foods, supplements, and other immunomodulatory products. Only studies that are published in reputable journals focused on product development or peer-reviewed scientific journals were considered. These studies must provide evidence of the product's effectiveness and safety. Research that does not meet these criteria or focuses on different plant species was excluded.

## 3 Taxonomic position of *Tinospora cordifolia*

*Tinospora cordifolia* is a member of the Menispermaceae family, which is part of the Ranunculales order in the Magnoliopsida class (commonly known as dicots) of the plant world. This classification includes it amid a wide range of blooming plants, while the Menispermaceae family is best recognized for its climbing vines and bushes. *Tinospora cordifolia*, often known as Guduchi or Gurjo, is an ancient medicinal plant from the Menispermaceae family of moonseeds [10]. The Menispermaceae family is abundant in tropical lowland environments, with 70 genera and 450 species. Tinospora is one of the most common genera in the Menispermaceae family, with around 15 different species [11]. *Tinospora cordifolia* is extensively widespread in Tropical India and may reach elevations of 1000 feet in South Asia, Indonesia, Philippines, Thailand, Myanmar, China, and Sri Lanka [12]. Ayurvedic and folk medicine systems frequently employ this compound, which may be found in a wide range of soil types (from acidic to alkaline) and only needs a small amount of soil moisture [13].

## 4 Plant description

*Tinospora cordifolia*, is a big, glabrous, perennial, deciduous vine with papery bark and juicy branches that spread widely. It is extensively distributed in Sri Lanka, Myanmar, and India [14]. Guduchi is indigenous to India's tropical areas, where it can survive in temperatures between 25 and 45 °C at elevations of up to 500 m. The leaves are heart-shaped, straightforward, and have a deep, vivid green hue. They have a broadly elliptical lamina that is 10–12 cm long and 8–15 cm broad, and they are alternating, stipulate, and whole [15].

The veins of leaves are multicostate and reticulated. The stems' surface has a completely studded appearance of warty tubercles. The skin's surface displays longitudinal fissures, with stems that have a diameter of 3–8 mm and a length of 3–5 cm. Large lenticels that resemble rosettes grow out of the succulent bark, which also includes deep clefts and spots [16]. The bark can be either grey or creamy white in hue. The branches give rise to aerial roots that are long and thread-like branches are either light greyish brown in hue or long and dingy white.

Grown on auxiliary and terminal racemes, the tiny, greenish-yellow blooms are unisexual. Female flowers often form single inflorescences, while male flowers are grouped [14,15]. Six sepals total, arranged freely in two sets of three each on each flower. In addition, six free, oval, membrane petals are smaller than the sepals.: Fruits develop in the winter (November) while flowers blossom in the summer (March to June) [15]. Fruits have a fleshy, orange-red hue and are composed of one to three ovoid, smooth droplets arranged in an aggregate on a stout stalk that has a subterminal-style scar. The moonseed family (Menispermaceae) is characterized by its bent seeds and embryo. Different decorations may be seen on the endocarp, or inner layer of the fruit wall [17,18].

## 5 Phytochemistry and nutritional composition

Giloy leaves are a potent source of nutrients and other essential elements. It was found that dehydrated giloy leaves contained high levels of calcium, protein, iron, crude fiber and ash as depicted in Table 1 [19]. All parts of *Tinospora cordifolia*, including leaves, stems, fruits, and roots, are used as functional foods. *Tinospora cordifolia* leaves are rich sources of nutrients, essential macronutrients, and micronutrients as shown in Table 1 [20,21].

**Table 1:** **Nutrient content of *Tinospora cordifolia* leaves per 100g** (Pandey et al.*,* 2016).

Nutrients	Fresh	Dehydrated	References
Moisture %	31.36	9.64	[[20], [19], [21]]
Ash %	2.3	5.880	
Carbohydrates (g)	3.34	7.53	
Protein (g)	2.30	5.23	
Fat (g)	0.36	1.05	
Fibre (g)	11.321	52.295	[20,21]
Iron (g)	5.87	22.55	
Calcium (g)	85.247	210	
Vitamin C (mg)	56	16	
Beta Carotene (μg)	303.7	428.5	
Energy (Kcal)	88.64	240	[20]

**Fig. 1:**
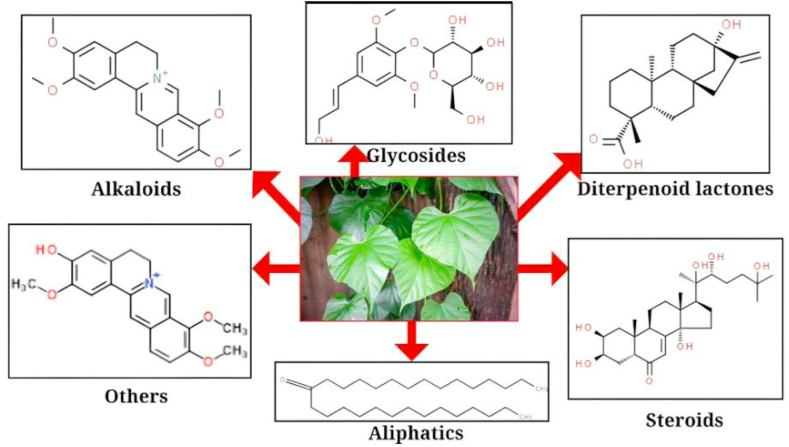
Bioactive components of *Tinospora cordifolia* (Giloy) leaves.

Giloy leaves are a potent source of bioactive compounds such as Alkaloids, Glycosides, Diterpenoids, and steroids as represented in Fig. 1.

## 6 Immunomodulatory activity of giloy leaves

**Fig. .2:**
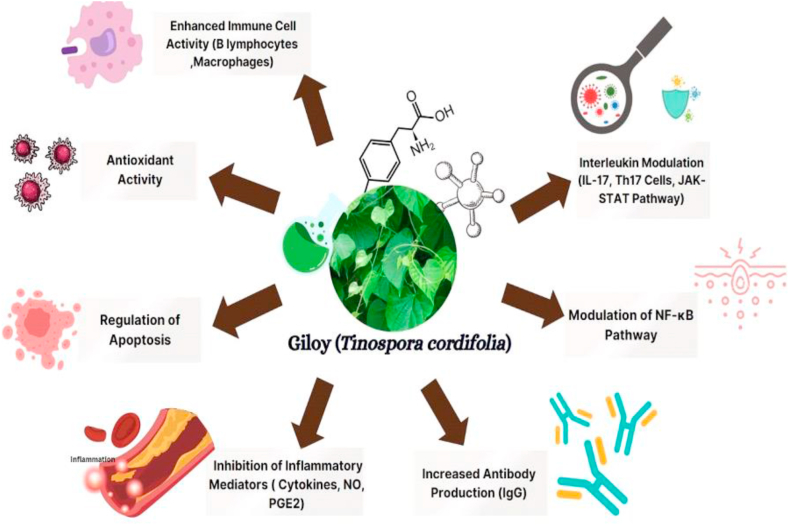
Immunomodulatory effects of giloy leaves.

The significant immunomodulatory qualities of *Tinospora cordifolia* leaves are attributed to a plethora of bioactive substances [22]. These active ingredients include alkaloids (found primarily in the stem) and steroids (beta-sitosterol, d-sitosterol, and g-sitosterol), as well as glycosides (18-norclerodane glycosides, furanoid diterpene glycosides, and tinocordiside) and palmatine D, choline D, tinosporine, magnoflorine, tetrahydropalmatine, and isocolumbin) [23,24]. Additional components found in its aerial sections include diterpenoid lactones (furanolactone, tinosporon, and columbin) as well as many other substances such cordifolioside A, 11-hydroxymuskatone, N-methyl-2-pyrrolidone, octacosanol, heptacosanol, and nonacosan-15-one [5,25]. Interestingly, the alkaloid palmatine is also present in the root. RR1, a polysaccharide extracted from the leaves, has been found to possess immune stimulatory properties and has the potential to be used as an adjuvant to boost immunity [26]. Additionally, the various bioactive ingredients present in giloy leaves affect the immune system by increasing cytokine synthesis, enhancing immune cell mitogenicity, and activating specialized immune cells like B cells and macrophages, which can trigger targeted immune responses [23], [27]. Further research into the exact mechanisms of these active ingredients will help to clarify the immunomodulatory potential of giloy leaves. The various mechanisms through which bioactive compounds of leaves help in boosting the immunomodulatory properties of the body have been illustrated in Fig. 2.

### 6.1 Cellular and molecular mechanism of immunomodulation

Giloy leaf extract has a high concentration of bioactive components such as alkaloids, glycosides, and terpenoids, contributing to its well-known immunomodulatory activities. The leaf extract constitutes bioactive components that stimulate a variety of immune cells, including natural killer (NK) cells, B cells, and T cells, resulting in the generation of favourable immuno-stimulating cytokines [[28], [29], [30]]. In addition, among HIV-positive patients, giloy leaves lower total leucocyte, neutrophil, and eosinophil counts [28]. These immuno-stimulating effects are linked to several bioactive chemical compounds found in the leaf, including 11-hydroxymuskatone, N-methyl-2-pyrrolidone, cordifolioside A, magnoflorine, tinocordioside, and Synringin [23]. These compounds specifically target immune cells present inside the human body such as B lymphocytes and macrophages, implying a regulated activation of the immune response [24,25]. This increases haemoglobin levels and polymorphonuclear leukocytes, indicating an overall increase in immune response [25]. These compounds are well known for their ability to promote a greater immune response by enhancing the proliferation and differentiation of lymphocytes. Thus, it increases total WBC, bone marrow cellularity and alpha esterase-positive cells in the marrow of bone which leads to an increase in humoral immune response. Some of the bioactive components appear to considerably raise IgG antibody levels, which are important components of the immunological response for complimentary inhibition of the pathway [31].

### 6.2 Inflammation and immunity boosting

Inflammation is an immune system response that is necessary for healing, but it can be harmful if it is severe or chronic. Herbal medicines such as giloy leaves have long been utilized to treat inflammation, however, worries about potential side effects have been highlighted [32]. It's important to note that while giloy (*Tinospora cordifolia*) has been used in Ayurvedic medicine for a long time, it should be used with caution due to potential negative effects, especially when taken in large amounts. Research indicates that exceeding the recommended dosage can lead to gastrointestinal issues such as nausea, upset stomach, and constipation [33]. Some studies conducted by researchers exhibits potential hepatotoxic effects due to Giloy's influence on liver enzymes and metabolism [21,33]. While the blood-sugar-lowering properties of giloy can be beneficial for diabetics, excessive use or concurrent use with blood-sugar-lowering medications can increase the risk of hypoglycemia (low blood sugar) [34]. Finally, allergic reactions are a possibility, particularly for individuals sensitive to plants of the Menispermaceae family [32]. These reactions may include skin rashes and swelling. Giloy leaf extract intake has been associated with the activation of phagocytic cells that aid in wound healing, as well as the acceleration of skin regeneration when applied topically to wounds and bruises. Giloy leaves' immunomodulatory properties, which have been explained by their bioactive chemical constituents such as alkaloids, glycosides, terpenoids, and polysaccharides, which contribute to their capacity to modulate immune responses and decrease inflammation [[35], [36], [37]].

**Fig. 3:**
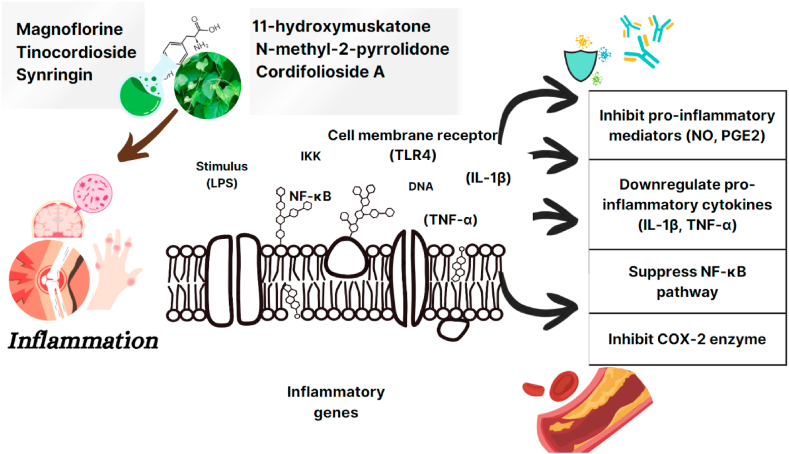
Lipopolysaccharide-induced inflammation and its attenuation by giloy leaf components through NF-kB pathway.

*Tinospora cordifolia* leaf extract encompasses bioactive chemical compounds that help to reduce inflammation and decrease immunological responses. The leaf extract contains a variety of metabolites, some of which have anti-inflammatory and antioxidant activities [38,39]. A molecule known as 7,9-Di-tert-butyl-1-oxaspiro (4,5) deca-6,9-diene-2,8dione was discovered as a potential dual inhibitor of COX enzymes, which is critical for treating inflammation. This chemical compound has been demonstrated high binding energies against COX 1 and COX 2, showing its potential as a drug like molecule for inflammatory therapy [38]. In addition, *T. cordifolia* leaf extract has been studied for in-vitro anti-inflammatory action and shown to decrease protein denaturation, a fundamental process in inflammation. These findings indicate that the chemicals in *Tinospora cordifolia* leaf extract produce anti-inflammatory actions and modify immunological responses by inhibiting COX and suppressing protein denaturation [38],[40]. Compounds in giloy leaves decrease inflammatory mediators, inhibit enzyme activity such as COX and alter immune cell function. Giloy leaves have the potential anti-inflammatory properties through a variety of pathways as shown in Fig. 3 about Inflammation and its action mechanism. More research will be required to determine their clinical benefits in controlling inflammatory diseases.

#### 6.2.1 Modulation of NF-κB pathway

Giloy leaves anti-inflammatory mechanisms that have been studied. According to studies giloy leaf extract prevented lipopolysaccharide (LPS)-stimulated macrophages from releasing prostaglandin E2 (PGE2) and nitric oxide (NO) [41]. Another study reported that giloy leaf extract reduced the expression of tumour necrosis factor-α (TNF-α) and interleukin-1β (IL-1β), two pro inflammatory cytokines in LPS-stimulated macrophages [24], [42]. Studies demonstrated by that giloy leaf extract suppressed the activation of the transcription factor-kappa B (NF-κB) signalling pathway, which is crucial for the inflammatory response [43]. Wilkinson showed that giloy leaf extract inhibited the activity of an enzyme produced by the human body cyclooxygenase-2 (COX-2), which plays a role in PGE2 synthesis [44].

These studies suggest that giloy leaves have multiple anti-inflammatory mechanisms, including the inhibition of NO and PGE2 production, the reduction of pro-inflammatory cytokine expression, and the suppression of NF-κB and COX-2 activity. These mechanisms make giloy leaves a promising natural anti-inflammatory agent with potential uses in the treatment of a variety of inflammatory conditions, which has been illustrated in Fig. 3.

### 6.3 Regulation of apoptosis in immune cells

Apoptosis is a process which helps in regulating the population of immune cells. For instance, after an immune response, excess immune cells may undergo apoptosis to prevent overactivity of the immune system. Apoptosis is used as a mechanism to eliminate infected cells [43]. When a cell is infected with a virus or bacteria and gets damaged or destroyed beyond repair, it can trigger apoptosis to prevent the spread of the infection [43], [45]. Some of the bioactive compounds of giloy leaves such as aporphine, magnoflorine, palmatine, tinocordiside, and cordifolioside -A, have demonstrated potential as anti-cancer agents [46,45]. They induce apoptosis and inhibit the growth of cancer cells, suggesting their significance in cancer therapy. Cancer cells undergo apoptosis, a process of planned cell death, when exposed to giloy leaf extract. It triggers nuclear condensation, the production of apoptotic bodies, and the activation of the essential apoptotic enzyme caspase-3 [47,48]. It also causes apoptosis in cancer cells by suppressing anti-apoptotic genes like Bcl-2 and upregulating pro-apoptotic genes like Bax (Bcl-2 associated X protein). Giloy leaf extract effectively inhibits cancer cell development by interfering with the G1 phase of the cell cycle [49], [50]. Cell division and DNA replication depend on this stage. Giloy leaf extract stops the cell cycle at this point, which stops cancer cells from proliferating and growing. Nitric oxide (NO) generation is enhanced by the stimulation of immune cells, specifically macrophages, by giloy leaf extract. NO causes cancer cells to become cytotoxic, leading to their destruction [51], [52].

### 6.4 Antioxidant

Giloy leaves contain several polyphenolic chemicals, including tannins, magnoflorine, jatrorrhizine, tembetarine, tinosporine, isocolumbin, palmatine, and tetrahydropalmatine. These substances are good candidates for antioxidant therapy because of their demonstrated potent antioxidant properties [35]. The giloy leaves extract's antioxidant capacity employs a range of in vitro tests. The extract exhibited noteworthy efficacy against DPPH, hydroxyl, and nitric oxide radicals, as demonstrated by the obtained results. The existence of energy-producing polyphenolic compounds that fight off free radicals and stop oxidative damage is the cause of this waste activity [23], [53]. The antioxidant activity of giloy leaf extracts in human lymphocytes against oxidative stress - driven through DNA damage. Studies have indicated that these extracts can mitigate DNA damage resulting from exposure to hydrogen peroxide, which is a strong inducer of oxidative stress [54].

This protection is due to the antioxidant capabilities of giloy leaves polyphenols, which remove free radicals and prevent DNA oxidation [53]. The effect of giloy leaf extract on oxidative stress and antioxidant state in rats with experimental liver injury [55]. Studies indicate that these extracts can enhance Antioxidant enzymes such as glutathione peroxidase, catalase, and superoxide dismutase as well as lower oxidative stress markers like malondialdehyde [56]. According to these results, giloy leaf extract may shield the liver from oxidative stress and liver damage [57]. The majority of the research indicates that the polyphenols present in giloy leaves have significant antioxidant properties and can be used to treat a variety of oxidative disorders associated with stress using antioxidant-based therapy.

### 6.5 Interleukin modulation

The immune-modulatory function of *Tinospora cordifolia* is associated with its bioactive compounds such as terpenes, glycosides, alkaloids, steroids, flavonoids, and polysaccharides [58]. IL-17, Th17 Cells, and the JAK-STAT Pathway interplay is crucial for understanding the potential immunomodulatory activity of Giloy leaf extract (*Tinospora Cordiofolia*). Proinflammatory cytokines like interleukin-17 (IL-17) are released by Th17 cells among other immune cells [59]. It is essential for protecting the body from infections, especially those caused by fungus and extracellular bacteria. Studies have shown that Autoimmune diseases and chronic inflammatory illnesses have been associated with elevated or dysregulated IL-17 generation [60].

T helper 17 (Th17) cells are a subset of CD4 + T lymphocytes that primarily secrete IL-17. Although they may contribute to autoimmune disorders, they are necessary for immune-mediated defence from external infections [61]. The differentiation and activation of diverse immune cells, including Th17 cells, are facilitated by the JAK-STAT pathway, a crucial signalling cascade [62]. It involves the phosphorylation of Janus kinases (JAKs) by cytokines attaching to particular cell surface receptors. Following JAK activation, STAT proteins (Signal Transducers and Activators of Transcription) relocate to the nucleus and regulate the expression of genes required for Th17 development and synthesis of IL17 [63].

Studies have shown that *Tinospora cordifolia* extract can reduce the amount of IL-17 generating cells in CD4^+^ T cells developed under Th17-polarizing circumstances suggesting an immunomodulatory effect [26]. Another study discovered that the water-soluble extract of *Tinospora cordifolia* improves macrophage phagocytic capabilities and significantly increases nitric oxide generation by stimulating splenocytes and macrophages at a dose of 1 mg/kg Research found that 100 μg/ml of *Tinospora cordifolia* extract caused 90 % cytotoxicity in B16F10 murine melanoma cells after 72 h of treatment [64].

Another research identified a polysaccharide from *Tinospora cordifolia,* RR1, which has been demonstrated to have innate immune stimulatory capabilities and can offer adjuvant-like action in the formation of a Th1-type immune response to an antigen [51], [65]. To put it in simple terms, *Tinospora cordifolia* activates the immune system by influencing cytokine synthesis, mitogenicity, and immune-effector cell activation, as well as enhancing phagocytic power and nitric oxide generation in macrophages [66]. The specific mechanism of interleukin modulation by which *Tinospora cordifolia* extract produces these benefits is unknown and will require more investigation and study.

## 7 Application of value-added products

Research on the uses of giloy (*Tinospora cordifolia)* leaf extract in various value-added products has revealed its significant immune-boosting properties and health benefits. Giloy extracts have been incorporated in different forms such as powder, fresh leaves, and stem juice to enhance the nutritional value and taste of the products which has been shown in detail in Table 2, Table 3. For instance, a combination of giloy leaf powder, besan, wheat flour, and adusa leaves was utilized to produce biscuits and sev, resulting in nutrient-rich products confirmed through tests like DPPH and Folin-Ciocalteu [67]. The phytochemicals present in giloy have been found to have a positive impact on the immune system, leading to increased consumption of homemade kadha (a herbal concoction) containing fresh giloy leaves and other ingredients during the COVID-19 pandemic [68]. Moreover, products like value-added cookies made with giloy and tulsi powders, herbal squash made with giloy leaf extract and pineapple juice, and herbal lassi made with giloy stem juice have displayed enhanced nutritional properties [69,70].

**Table 2:** Immunomodulatory giloy leaves-based value-added products.

S. No.	Objective of study	Value added Product	Overview of Study	Reference
**1.**	Evaluation of Herbal Leaves for the Development of Value-Added Food Product	Biscuit and Sev	In this study, the giloy leaves were used to make value-added products such as biscuits and sev. The greatest method to add medicinal plants' nutritional advantages to humans' everyday diets to help fight degenerative illnesses is to provide value to food items by including them. It shows that the nutrients in these products can help in the fight against diseases and improve immunity.	[67]
**2.**	Consumption of natural products and Ayurvedic decoctions “Kadha” as immunity-boosting measures during the spread of COVID-19 in Delhi	Ayurvedic Decoction “Kadha”	This study aimed to explore the prevalence of consumption of natural products and Ayurvedic decoctions “kadha” as immunity-boosting measures during the initial phase of the COVID-19 pandemic. Around 540 responses were taken through an online survey which shows the usage of immunity-boosting measures and Ayurvedic decoctions “kadha” among the adult residents of Delhi belonging to different age groups was effective.	[68]
**3.**	Development of Giloy leaves-based Herbal Squash incorporated with Pineapple	Giloy leaves based Herbal Pineapple Squash	This research work was an effort in the direction of producing value-added instant beverages. Due to the functional and nutritional goodness of both plants, it can be further exploited in the development of healthy beverage products to develop giloy leaves-based herbal squash incorporated with pineapple and to determine its physical and biochemical properties.	[69]
**4.**	Development of value-added cookies supplemented with giloy leaves and Tulsi powder	Cookies with giloy leaves and tulsi powder	In this study, giloy leaves powder was utilized in place of whole wheat flour to create herbal biscuits. When preparing herbal cookies, whole wheat flour partially replaces the powdered tulsi leaves and giloy leaves stems.	[71]

**Table 3:** Traditional Giloy leaves Based Value-added Products.

S. No.	Objective of study	Value-added Product	Overview of Study	Reference
**1.**	Development of herbal lassi using giloy leaves stem juice	Herbal lassi	Based on the current study, giloy leaves may be utilized to successfully make herbal lassi. Based on microbiological characteristics, the produced lassi was deemed safe for consumption. Natural antioxidants, such as giloy leaves, effectively lower the risk of heart disease, cancer, and many inflammatory processes, and have a positive impact on cardiovascular illnesses. It also strengthens immunity.	[70]
**2.**	Development of Antioxidant-Rich Herbal Tea Bags	Herbal Tea Bags with Giloy leaves	This study was conducted to develop herbal tea bags using giloy leaves and different herbs which can provide antioxidant properties, boost immunity and also enhance the efficacy of white blood cells which helps in fighting against infections and bacteria-causing diseases.	[72]
3.	Value Addition and Fortification in NonCentrifugal Sugar (Jaggery): A Potential Source of Functional and Nutraceutical Foods	Jaggery fortified with giloy leaves.	Jaggery is classified as a nutraceutical since it contains a range of vital amino acids, antioxidants, phenolics, minerals (calcium, phosphorus, iron), and vitamins. Jaggery is a more natural source of nutrients for health benefits and might be utilized as a healthier nutritional option for white sugar. Giloy leaves are incorporated as a health-supporting herb to generate even superior antioxidant, detoxifier, digestive, and immune booster products with potential functional and nutraceutical value.	[73]
**4.**	Formulation of Herbal Candies Containing Giloy Leaves Satva: A Nutritious and Palatable Herbal Confectionery Option	Herbal Candies Containing Giloy leaves	The addition of giloy leaves satva in confections shows potential for improving immunity as it fights free radicals and boosts the body's mechanisms for defence against infections. It offers an intriguing discipline of study and innovation, providing a natural and pleasant method for immunomodulation	[74]
**5.**	Sensory Evaluation of Laddu Enriched with Giloy leaves (*Tinospora* Cordifolia) - Iron-Rich Powder	Laddu Enriched with Giloy leaves	The purpose of this study is to formulate and establish a standard giloy leaves laddu for people with anaemia and to determine its level of acceptability. The goal is to make a healthy laddu and put giloy leaves powder into it.	[75]
**6.**	A study on incorporation of giloy leaves for the development of shelf-stable goat milk-based functional beverage	Goat milk incorporated with giloy leaves	By combining debittered giloy leaf juice with goat milk, a study was carried out to develop a shelf-stable giloy leaves goat milk beverage. The product profile was examined in depth, including its proximate composition, bioactive qualities, sensory, rheological, and structural characteristics. The addition of giloy leaves in this creates a functional beverage with a longer shelf life that targets health issues and boosts immunity.	[76]

The research has shown that giloy can be used in a plethora of products. The sensory qualities of giloy leaves, tulsi, and ginger herbal tea bags were optimised [72]. Additionally, herbal candies made from giloy stems juice and sugar offer a nutritious and nutrient-dense option [74]. Fortified jaggery with giloy leaves powder has shown enhanced nutritional profiles, and shelf-stable goat milk enriched with giloy stem juice has demonstrated extended shelf life and stability [73,76]. Furthermore, giloy powder has been used in giloy laddus, where it is combined with other components [75]. These diverse uses highlight Giloy's leaf extract potential to improve the nutritional value and health-promoting properties of food items.

### 7.1 Comparison between traditional and modern value-added products

While traditional methods do not allow any combination of other ingredients, modern methods often combine giloy leaves with other herbs to enhance its effect for specific health concerns like stress, skin health, joint pain etc. Quality assurance varies in both traditional and modern value-added products, with modern manufacturing practices there is more focus on quality control and standardized formulations to ensure consistency and quality control in Giloy leaves-based products.

Different Varieties of modern value-added products are available nowadays, while traditional value-added products are very limited as shown in Table 3, Table 4. Table 3, Table 4 illustrate the contrast between traditional and current applications of giloy leaves. Table 4 displays the Giloy-based products available in the market, while Table 3 outlines traditional uses. There are various value-added products in today’s modern era like capsules, juice, giloy leaves powder, giloy leaves extract and many more while traditional value-added products were limited to most consumers where churan, and kadha (juice blend) are common among all. Modern products are fortified with different vitamins and minerals to enhance the nutritional value of the product while traditional products are not fortified and produced as it is consumed. Modern giloy leaves products are widely available in pharmacies, local stores and online platforms, and are greatly accessible to consumers worldwide, which is not the same in the case of traditional value-added products. Modern giloy leaves products offer a convenient form of consumption, making it easier for individuals to consume and incorporate into their daily routines plus they are less time-consuming. Modern giloy leaves products have a high shelf life as they are preservatives with chemicals like sodium benzoate and potassium sorbate etc. or with various modern preserving methods. Traditional giloy leaves products typically use natural and herbal ingredients plus no chemicals were used resulting in less shelf life of the product. Traditional value-added products are made by methods that are passed down from generation to generation. Which involves manual labour, the use of natural ingredients and traditional equipment, which helps in preparing the perfect desired product. In Modern value-added products, advanced technologies and machines are used for production which works on standardized methods. Automatic machines, standard measurements, and quality control techniques are very common for preparing required products.

**Table 4:** Commercially giloy leaves based on value-added products.

Product Name			Brand Name		Health Claims		Ingredients	Reference
Capsules/Tablets rowhead								
**1.**	Guduchi Immunity Wellness		Himalaya		● Strengthens Immunity ● Fights infections ● Supports detoxification ● Helps in increasing the performance of white blood cells		Giloy leaves Stem Extract	https://www. amazon. in/HimalayaWellness-Herbs-Guduchi-Immunity/dp/B00B8ROM1M
2.	Giloy leaves Immunity Booster		Zandu		● Protection Against Infections & Immunity Booster. ● Promotes Liver Health ● Stress Relief ● Anti-oxidant Compounds for Good Skin Health.		Giloy leaves Stem Extract	https://www. amazon. in/ZanduGuduchi-herbs-immunitywellness/dp/B07V6TPRQ8
**3.**	Guduchi (Giloy leaves) Ghanbati		Baidyanath		● Boost Immunity ● Reduces anxiety and improves mental strength. ● Rich in antioxidants and has anti-allergic, antifungal & anti-bacterial benefits. ● Maintain strength and vitality ● Aids pitta disorders & improves digestion.		Giloy leaves Stem Extract. Ashwagandha Amla Gorakhmundi	https://www. amazon. in/Baidyanath-Guduchi-Giloy leaves-GhanBati/dp/B08CY425YB
4.	Divya Giloy leaves Ghanvati		Patanjali		● Immune boosting ● Antioxidant properties and anti-inflammatory effects. ● Provide liver support ● Promote respiratory health.		Giloy leaves Stem Extract	https://www. amazon. com/Patanjali-Giloy leaves-Ghan-Vati-Tablets/dp/B07HWTJ2G3
**5.**	Jiva Giloy leaves		Jiva		● Boosts Immunity. ● Helps fight respiratory problems. ● Helps reduce joint pain. ● Improve digestion. ● Helps in detoxification.		Giloy leaves Stem Extract	https://www. amazon. in/Jiva-Giloy leaves-Capsule-Respiatory-Detoxification/dp/B093KTSXX8
**Powder** rowhead								
1.		Organic Giloy leaves/Guduchi Stem Powder		CARMEL ORGANICS	● Boosting the immune system. ● Improving digestion. ● Reducing stress and anxiety. ● Controls blood sugar level.	Giloy leaves stem powder		https://www. amazon. in/CertifiedAmruthavalli-Tinospora-cordifolia-Preservative/dp/B08CHGD429
**2.**		Natural Organic Guduchi Powder/Giloy leaves Powder		MY HERB	●Aids in detoxification, increases antibodies, and stimulates vitality. ●Relieves stress and anxiety while also replenishing the body. ●Combats illnesses related to respiration. Demonstrates anti-inflammatory and antiarthritic activities	Giloy leaves stem powder		https://www. amazon. in/GuduchiPowder-Tinospora-cordifoliaMetabolism/dp/B08MTZPL1C
**3.**		Giloy leaves Satva		Baidyanath	●Useful in treating burning sensation in hands & feet, headache, metallic taste in mouth & excessive thirst. ●Enhances immunity of the body.	Giloy leaves extract powder		https://www. amazon. in/Baidyanath-Giloy leaves-Satwa-Pack-140/dp/B08KT8VB8P
**Teas**		Moringa & Giloy leaves Green Tea with Lemongrass		Care	●Support to build an Immune system. ●Make bones and joints healthy. ●Help in losing weight. ●Enhance metabolism.	Moringa Giloy leaves Licorice Ginger Lemongrass		https://www. amazon. in/Moringa-Lemongrass-Immunity-Management-Ayurvedic/dp/B0836MJH7G
**2.**		Giloy leaves Immunity Tea		Jiva	●Effective immunity booster. ●Improve skin condition and reduce acne and pimples. ●Helps with seasonal colds and coughs. ●Helps to improve overall health & well-being.	Giloy leaves stem powder		https://www. amazon. in/Jiva-Giloy leaves-150gm-Pack-Immune-Tea/dp/B085S4ZFZQ
**3.**		Giloy leaves Tea		One Herb	●Immunity booster. ●Improves digestion. ●Helps reduce skin infections. ●Relieves chronic fever. ●Help regulate blood sugar levels. ●Boosts metabolism and aids weight management.	Giloy leaves stem powder		https://www. amazon. in/One-Herb-Ultimate-Immunity-Infections/dp/B08VNLYKTW
**4.**		Ayurvedic Herbs Giloy leaves Green Herbal Tea		Rishtpusht	●Immunity booster. ●Helps maintain a healthy heart and nervous system. ●Fight allergies and common infections. ●It improves blood circulation and eliminates harmful substances from the body.	Giloy leaves Satva, Dry ginger, Clove, Cardamom, Lemon Basil, Green tea, Cumin seed, Sugar.		https://www.amazon.in/Rishtpusht-Ayurvedic-Immunity-Booster-Natural/dp/B091YTDSK8
Juice rowhead								
**1.**		Giloy leaves juice		Dabur	●Natural immunity booster ●Antioxidant properties ●Good for liver and skin health ●Natural detoxifier	Giloy leaves extract		https://www. amazon. in/DABUR-Giloy leaves-Neem-JuiceTulsi/dp/B087DJ9L3K
**2.**		Giloy leaves tulsi juice.		Vansaar	●Cleans and detox the gut ●Boost immunity & fight against common cough cold ●Antioxidant ●Anti-microbial ●Anti-bacterial ●Anti-inflammatory	Giloy leaves stem Tulsi leaves		https://www. amazon. in/Enhancing benefits-Handpicked-all-round-Immunity/dp/B07CYYPFFR
3.		Giloy leaves juice		Baidyanath	●Boost immunity ●Rich source of antioxidant ●Helps maintain a healthy heart and nervous system	Giloy leaves extract		https://www. amazon. in/Baidyanath-Boost-Immunity-Natural-Giloy leaves/dp/B087LTSC5M
**4.**		Wild tulsi and giloy leaves juice		Krishna’s herbal & ayurveda	●Helps regulate blood sugar levels. ●Boost metabolism & improve digestion ●Stimulates insulin secretion ●Helps manage weight	Giloy leaves, Jamun, Bel Patra, Amla, Methi, Karela, Kutki, Vijaysar, Tulsi, Gudmaar Neem		https://www. amazon. in/Krishnas-Herbal-Ayurveda-GeloyTulsi/dp/B07PLYBMMB
**5.**		Giloy leaves vital		AVG health organics	●Immunity booster ●Anti-pyretic ●Reduces inflammation & pain in the body ●Glucose metabolism	Giloy leaves Ashwagandha Basil Amla berry		https://www. amazon. in/AVGHealth-Organics-Ashwagandha-Premium/dp/B08CYCPTV6

These methods are often more precise and accurate as compared to traditional techniques which require a large amount of labor work. Traditional value-added products are higher in cost as compared to modern products as they have higher production costs which include labor-intensive processes, limited production scale, and the use of expensive and premium quality ingredients. As a result, they are often highly-priced. Modern products benefit from modern techniques, machines, high-scale production processes, and standardized measurement of ingredients. Which often leads to lower production costs and reasonable pricing.

Traditional value-added products also lack innovation as they prioritize traditional or hereditary methods which lack creativity and innovation, resulting in passing down the methods and recipes without any modifications. In the case of modern value-added products modifications are done with time, and new researches are done resulting in new recipes, new flavours, new techniques etc. Traditional production methods have limitations in organizing due to their reliance on manual labour and different techniques. Increasing production volumes is also challenging without compromising quality and effectiveness.

While Modern production methods are highly organized due to automation, Standardization and efficient manufacturing processes. Companies can easily adjust production volumes to meet different demands without sacrificing the quality and effectiveness of the product. Traditional products often target markets or consumers seeking authenticity, and local produce. They may be available through speciality stores or directly from the production area. Traditional products use less marketing while Modern products appeal to a broader consumer base due to their convenience, and wider distribution channels. They are often spread in supermarkets, online platforms, and large retail chains.

## 8 Safety and toxicity

*Tinospora cordifolia*, also known as Giloy, is a revered medicinal plant in the Indian Ayurvedic tradition, renowned for its diverse therapeutic applications [77]. However, recent studies have raised concerns about the potential hepatotoxic effects associated with the consumption of giloy leaves powder. The traditional use of giloy in Ayurveda has been well-established, with the plant being recognized for its anti-pyretic, anti-inflammatory, and hepatoprotective properties. However, a growing body of evidence suggests that prolonged or excessive consumption of Giloy leaves powder may lead to liver damage, a phenomenon known as hepatotoxicity.

Several in vitro and animal studies have investigated the potential hepatotoxic effects of giloy. Researchers have observed that exposure to giloy extracts can result in increased lipid peroxidation, lactate dehydrogenase release, and a decline in glutathione-S-transferase activity, all of which are indicative of cellular stress and potential liver injury [49]. Alkaloids (berberine, palmatine, and jatrorrhizine) and sinapic acid are believed to contribute to its hepatoprotective effects. Berberine, for instance, has been shown to reduce inflammation by inhibiting TNF-α-mediated proinflammatory pathways and nitrosative stress by suppressing iNOS activity [78]. Beyond its hepatoprotective properties, Tinospora cordifolia exhibits a broad spectrum of biological activities, including anticancer, anti-inflammatory, antimicrobial, and antioxidant effects. It is generally considered safe at doses up to 2000 mg/kg. The pharmacological actions of Tinospora cordifolia are attributed to a diverse array of phytochemicals, including polyphenols, alkaloids, steroids, terpenoids, and glycosides [78,79]. Hepatotoxicity, or liver damage, can be caused by a variety of factors, including the ingestion of toxic substances, overdose of medications, and even certain herbal preparations [79].

## 9 Future prospective

Although information on the immunomodulatory qualities of giloy leaves is growing, there is still a significant lack of understanding about the precise processes behind these effects. The precise molecular pathways and cellular interactions underlying the observed antioxidant activity, inflammation modulation, Th17 cell regulation, inhibition of the NF-κB pathway, interleukin modulation, and immunosuppressive and white blood cell-enhancing properties remain unclear. To understand the full therapeutic potential of giloy leaves, it is imperative to close this gap between data and intricate processes. Therefore, future research on several areas should be done including important signalling pathways like NF-κB, which have previously been connected to giloy leaves effects, should be the focus of research. These processes have to be clearly understood. Targeted therapies need a clearer comprehension of the molecular interactions that components of giloy leaves have with these pathways. Extensive research is required to determine the particular pathways through which giloy leaves constituents interact with other types of immune modulatory cells, such as Th17 cells, macrophages, and natural killer cells. Having a thorough understanding of how giloy leaves affect these cells' activation, differentiation, and function would help develop therapeutic applications. Investigate the possible benefits that giloy leaves may have when combined with other widely consumed foods that are high in vitamins, minerals, or antioxidants. This can help with dietary suggestions for enhancing immune function and for nutraceutical development. Examine the impacts of food matrices and determine which food ingredients can improve the bioavailability of the bioactive chemicals found in giloy leaves. This information can help formulate products in a way that maximizes health benefits. Contrast the immunomodulatory properties of giloy leaves with known immunomodulatory medications or natural compounds. This analysis can shed light on the distinct processes of giloy leaves and discover potential synergies in combination therapy. Through establishing a connection between current data and comprehensive research, scientists can fully realize the potential of giloy leaves as a medicinal agent. This all-encompassing strategy will open the door for focused therapies and enhanced formulations for particular immune-related ailments. In Future researchers should prioritize the development of standardized protocols for giloy leaves to ensure consistent quality and efficacy. Further research is necessary for negotiating the regulatory environment for giloy-based applications in order to commercialize the product.

## 10 Conclusion

In conclusion, the study we did on the immunomodulatory properties of giloy leaves states that they have many health benefits such as boosting immunity, regulating apoptosis in immune cells and acting as antioxidants. They have active compounds such as alkaloids, polysaccharides, flavonoids, and terpenes present in them that help in immune cell activation which stops cell damage and immune cells like macrophages and B cells which help in inflammation reduction and stop cancer cells from increasing. Giloy leaves modulate interleukins and Th17 cells which helps in protection against infections and increasing immunity.

Many modern products are now available in the market that are adapted, formulated and fortified from traditional uses of giloy leaves which can help in treating immunity-related disorders and other diseases. However, for better application and usage of this, further research and studies are required to understand its mechanisms which further helps in understanding its effects, applications and how they work with other compounds and treatments to enhance its use. Giloy leaves contain active ingredients that may be responsible for these benefits. Convenient options to include Giloy into daily routines are provided by modern products. It is crucial to recognize that there may be interactions between giloy leaves and specific drugs. Therefore, we advise speaking with a healthcare professional before incorporating giloy-based products into daily routine to ensure safe and effective use, particularly if you are taking any medications.

Overall, giloy leaves are said to be a natural immunomodulatory agent having many other properties such as antioxidant, hepatoprotective, anti-inflammatory and many more which required the researchers to go further to understand its properties fully.

## CRediT authorship contribution statement

**Jyoti Singh:** Writing – review & editing, Writing – original draft, Conceptualization. **Etika Saxena:** Writing – original draft. **Anjali Raj Chaudhary:** Writing – review & editing. **Mandeep Kaur:** Writing – review & editing. **Molly Salotra:** Writing – original draft, Supervision. **Prasad Rasane:** Writing – review & editing, Writing – original draft. **Sawinder Kaur:** Writing – review & editing, Supervision. **Sezai Ercisli:** Writing – review & editing, Methodology, Investigation. **Melekber Sulusoglu Durul:** Writing – review & editing, Supervision, Formal analysis. **Mehmet Ramazan Bozhuyuk:** Writing – review & editing, Resources. **Ahmet Hakan Urusan:** Writing – review & editing, Software. **Riaz Ullah:** Writing – review & editing, Resources.

## Ethics declaration

This study did not require informed consent or review and approval by an ethical committee because it was a literature analysis that solely used data from published studies and did not involve any direct experimentation or studies on living beings.

## Data availability statement

The research reported in the paper did not involve the utilization of any data. This article's accompanying data has not been added to any publicly accessible databases.

## Funding

This study received no explicit financing from public, commercial, or non-profit organizations.

## Declaration of competing interest

The authors declare that they have no known competing financial interests or personal relationships that could have appeared to influence the work reported in this paper.
